# B-Cell Lymphoma 2 (Bcl-2) and Regulation of Apoptosis after Traumatic Brain Injury: A Clinical Perspective

**DOI:** 10.3390/medicina56060300

**Published:** 2020-06-18

**Authors:** Hansen Deng, John K. Yue, Benjamin E. Zusman, Enyinna L. Nwachuku, Hussam Abou-Al-Shaar, Pavan S. Upadhyayula, David O. Okonkwo, Ava M. Puccio

**Affiliations:** 1 Department of Neurological Surgery, University of Pittsburgh Medical Center, Pittsburgh, PA 15213, USA; Zusman.Benjamin@medstudent.pitt.edu (B.E.Z.); Nwachukuel@upmc.edu (E.L.N.); aboualshaarh@upmc.edu (H.A.-A.-S.); okonkwodo@upmc.edu (D.O.O.); puccam@upmc.edu (A.M.P.); 2 Neurotrauma Clinical Trials Center, University of Pittsburgh Medical Center, Pittsburgh, PA 15213, USA; 3 Department of Neurological Surgery, University of California San Francisco, San Francisco, CA 94110, USA; john.yue@ucsf.edu; 4 Brain and Spinal Injury Center, Zuckerberg San Francisco General Hospital, San Francisco, CA 94110, USA; 5 Department of Neurological Surgery, University of California Diego, San Diego, CA 92093, USA; pavan8632@gmail.com; 6 Department of Neurological Surgery, Columbia University Medical Center, New York, NY 10032, USA

**Keywords:** Bcl-2, Bax, Bcl-x_L_, apoptosis, programmed cell death, traumatic brain injury

## Abstract

*Background and Objectives:* The injury burden after head trauma is exacerbated by secondary sequelae, which leads to further neuronal loss. B-cell lymphoma 2 (Bcl-2) is an anti-apoptotic protein and a key modulator of the programmed cell death (PCD) pathways. The current study evaluates the clinical evidence on Bcl-2 and neurological recovery in patients after traumatic brain injury (TBI). *Materials and Methods:* All studies in English were queried from the National Library of Medicine PubMed database using the following search terms: (B-cell lymphoma 2/Bcl-2/Bcl2) AND (brain injury/head injury/head trauma/traumatic brain injury) AND (human/patient/subject). There were 10 investigations conducted on Bcl-2 and apoptosis in TBI patients, of which 5 analyzed the pericontutional brain tissue obtained from surgical decompression, 4 studied Bcl-2 expression as a biomarker in the cerebrospinal fluid (CSF), and 1 was a prospective randomized trial. *Results:* Immunohistochemistry (IHC) in 94 adults with severe TBI showed upregulation of Bcl-2 in the pericontusional tissue. Bcl-2 was detected in 36–75% of TBI patients, while it was generally absent in the non-TBI controls, with Bcl-2 expression increased 2.9- to 17-fold in TBI patients. Terminal deoxynucleotidyl transferase-mediated biotinylated dUTP nick-end labeling (TUNEL) positivity for cell death was detected in 33–73% of TBI patients. CSF analysis in 113 TBI subjects (90 adults, 23 pediatric patients) showed upregulation of Bcl-2 that peaked on post-injury day 3 and subsequently declined after day 5. Increased Bcl-2 in the peritraumatic tissue, rising CSF Bcl-2 levels, and the variant allele of *rs17759659* are associated with improved mortality and better outcomes on the Glasgow Outcome Score (GOS). *Conclusions:* Bcl-2 is upregulated in the pericontusional brain and CSF in the acute period after TBI. Bcl-2 has a neuroprotective role as a pro-survival protein in experimental models, and increased expression in patients can contribute to improvement in clinical outcomes. Its utility as a biomarker and therapeutic target to block neuronal apoptosis after TBI warrants further evaluation.

## 1. Introduction

Traumatic brain injury (TBI) is a leading cause of mortality and disability worldwide, and in the US, it leads to an estimated 3 million annual emergency department (ED) visits and 56,000 deaths [1,2]. The diagnosis, treatment, and prognostication of TBI using the armamentarium that is currently available to the clinician remain hindered by the heterogeneity of traumatic pathologies. The incorporation of adjunct diagnostic tools has been shown to improve clinical assessment and risk stratification [3,4]. Knowledge of the biomolecular changes as a consequence of the mechanical impact of TBI, particularly the regulatory pathways of apoptosis and neuroinflammation, can improve the current understanding on secondary injury burden with the goal of identifying additional targets of optimal treatment [5,6].

Neuro-intensive strategies entail advanced modalities of intracranial monitoring to detect and manage intracranial hypertension, cerebral edema, hypoxia, and metabolic disequilibrium. There is growing interest in the TBI literature to further elucidate B-cell lymphoma 2 (Bcl-2), a pro-survival protein in neuronal tissue, as a potential diagnostic and therapeutic target in the acute period post-injury [7]. Experimental traumatic and ischemic models in rodents have indicated that the Bcl-2 family has key modulators of programmed cell death [8,9]. Bcl-2 protein is upregulated in neurons that are injured but could survive, while it is absent in cells with apoptotic morphology [10].

In human subjects, evidence of the biomolecular changes in Bcl-2 expression and apoptotic suppression are less clear. Current challenges include methodological limitations associated with patients, TBI heterogeneity, and clinical practice variability across institutions. Herein, we provide a summary of the relevant data on Bcl-2 in human research, its association with cellular response to injury and apoptosis in the acute period, and long-term clinical outcomes at follow-up.

## 2. Materials and Methods

### 2.1. Study Selection

A comprehensive and systematic search was conducted in the National Library of Medicine PubMed database for all human studies published on Bcl-2 in the setting of head injury. The search terms ((B-cell lymphoma 2[Title/Abstract]) OR (Bcl-2[Title/Abstract]) OR (Bcl2[Title/Abstract])) AND ((brain injury[Title/Abstract]) OR (head injury[Title/Abstract]) OR (head trauma[Title/Abstract]) OR (traumatic brain injury[Title/Abstract])) AND ((human[Title/Abstract]) OR (patient[Title/Abstract]) OR (subject[Title/Abstract])) were utilized to query the existing literature in the English language. Primary literature on humans, excluding animal transgenic models, was included in the current review. Study authors independently reviewed each article and determined its relevance, and all articles were unanimously included or excluded based on the established set of inclusion and exclusion criteria.

### 2.2. Inclusion and Exclusion Criteria

Studies were included if they were investigations of Bcl-2 involving human cortical tissue, neurons, or biomarkers in the setting of traumatic brain injury or head injury, specifically to elucidate apoptosis and cellular response to injury following the initial injury. References from the study selection were reviewed and included if these criteria were met and unanimously agreed upon by the authors. The exclusion criteria were if studies were not in the English language, were animal or transgenic research, lacked substantial data on Bcl-2, or were review papers.

### 2.3. Literature Search Summary

**Figure 1:**
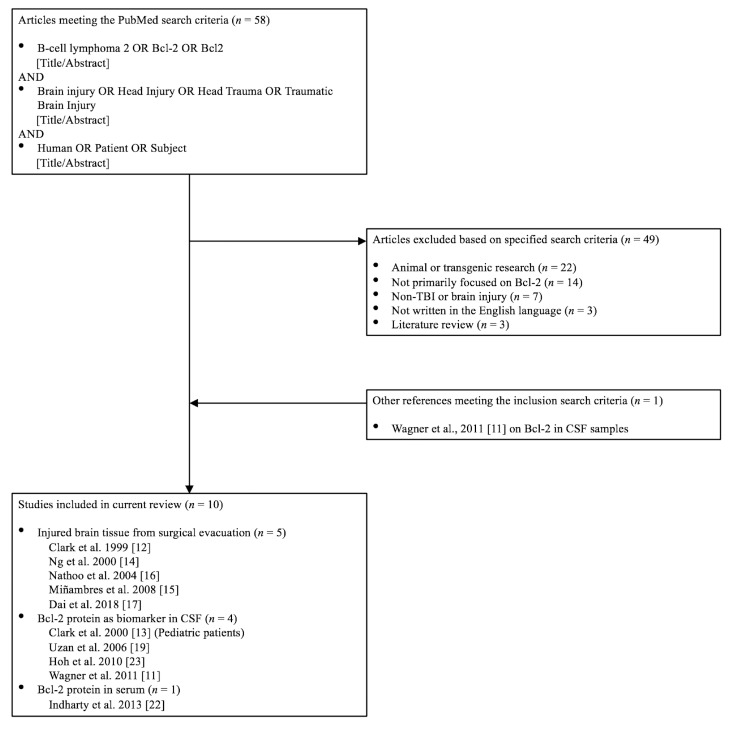
Flow chart indicating the search criteria and the included articles. Bcl-2: B-Cell Lymphoma 2; TBI: Traumatic brain injury; CSF: cerebrospinal fluid.

A total of 58 investigations were reviewed based on the initial search, of which 9 were selected for inclusion (Figure 1). Forty-nine investigations were excluded for the following criteria: animal or transgenic research (*n* = 22), the primary focus was not on Bcl-2 (*n* = 14), not related to head injury (*n* = 7), in non-English language (*n* = 3), and literature review (*n* = 3). One study, Wagner et al. [11], was added after evaluation of relevant citations. Therefore, a total of 10 studies were deemed fit for inclusion in this systematic review. There are 5 cohort studies that utilized injured brain tissue from TBI patients, 4 cohort studies involving Bcl-2 protein as a biomarker from cerebrospinal fluid (CSF), and 1 prospective randomized trial. A summary of the included studies and their important findings are provided in Table 1. With the exception of one investigation of pediatric patients, the rest were adult patients with TBI.

## 3. Results

### 3.1. Histological Evidence of Bcl-2 Expression Following TBI

Five studies have assessed ex-vivo human cortical tissue for evidence of Bcl-2 expression following traumatic brain injury. The first study to investigate the role of Bcl-2 in apoptosis following TBI was published by Clark and colleagues in 1999 [12]. They studied tissue from a cohort of 8 adult TBI patients who underwent decompressive surgery. Six of the eight patients (75.0%) were male and had a median admission Glasgow Coma Scale (GCS) score of 5.5 (range 3–15). Pericontusional cortex was taken during surgery (6/8 on injury day 1, range day 1–9) and was compared against post-mortem cortical samples from 6 adult controls who died of non-TBI related causes. All TBI patients had evidence of increased Bcl-2 expression by western blot compared to only one control patient (16.7%) who had evidence of Bcl-2 expression (17-fold increase, *p* = 0.020). The one control patient had died of cardiac arrest and was likely to have experienced cerebral ischemia as a reason for Bcl-2 upregulation [7]. This was in contrast to Bcl-2 family members Bcl-x_L_ and Bax, which were similarly expressed in both TBI patients and controls. When sections from 4 TBI patients and 4 controls were examined with immunofluorescence, the investigators found that 75.0% of TBI patients, but none of the controls, were Bcl-2-positive [12], whereas terminal deoxynucleotidyl transferase-mediated biotinylated dUTP nick-end labeling (TUNEL)-positive cells demonstrating DNA fragmentation were seen in most TBI tissues and 2 out of 6 (33.3%) of the controls. The association between histological findings and outcome was not reported.

Clark and colleagues also provided the first histological evidence of Bcl-2 expression in a pediatric TBI cohort [13]. As part of a larger study on CSF levels of apoptotic markers in pediatric TBI patients, Clark et al., histologically assessed pericontusional tissue from two patients who underwent decompressive surgery for refractory intracranial hypertension. Both patients had initially presented with a GCS of 3. One was a three-year-old who underwent surgery on post-trauma day 12. The other was an 11-year-old who underwent surgery on post-trauma day 1. The investigators found that both patients had evidence of Bcl-2 expression by western blot, though the three-year-old exhibited a much more robust band. This also correlated to higher average CSF Bcl-2 over the first three days post-injury. These samples were compared to control tissue from adult samples, which again showed no evidence of Bcl-2 expression, but no pediatric controls were available [7]. Findings from the CSF analysis are explained in the next section.

Shortly after Clark and colleagues published their first study, Ng et al., investigated Bcl-2 expression in pericontusional tissue from adult severe (GCS < 9) TBI patients [14]. Ng and colleagues examined brain tissue specimens from 11 patients after excluding contusional core samples: 8 of the 11 (72.7%) patients were male and went to surgery between 2 and 192 h from injury. Expression of Bcl-2 by immunohistochemistry (IHC) showed that Bcl-2 was expressed in 6 of the 11 (54.5%) samples. They found that all 6 of the patients who were Bcl-2-positive were alive at six months, while only 1 of the 5 Bcl-2-negative patients survived (*p* = 0.01). There was also a trend towards lower ICP in the 12 h post-surgery in the Bcl-2-positive group (13.5 ± 3.7 versus 40.8 ± 20.8 mmHg, *p* = 0.057), though this difference did not reach statistical significance. TUNEL-positive cells were detected in 8 of the 11 (72.7%) TBI patients. Age, sex, and initial GCS did not differ between Bcl-2-positive and negative patients [14].

Miñambres and colleagues found similar results in a cohort of 11 adult severe TBI patients (72.7% male) that included tissue taken both during decompressive surgery and post-mortem at autopsy after excluding patients who died within 48 h of injury [15]. Four of eleven (36.4%) injured tissue samples were obtained during surgery, and seven samples were obtained at autopsy. All 4 patients in the craniotomy group had a good outcome at 6 months (Glasgow Outcome Scale (GOS) 4 or 5). The time from admission to surgery ranged from 11 to 51 h. At autopsy, tissue was taken both from the pericontusional region and from well-preserved tissue distal to the site of injury. The authors did not note time from admission to death, but time from death to autopsy ranged from 9 to 16 h. These 11 patients were compared to 5 adult controls (80.0% male) who died from non-cranial causes. Time from death to autopsy ranged from 12 to 16 h in the controls. Expression of Bcl-2 was assessed semi-quantitatively by IHC on a 0–3 scale by two observers blind to clinical status. They found that Bcl-2 expression was positive in 3 of 4 (75.0%) TBI patients who underwent craniotomy, 1 of 7 (14.3%) post-mortem TBI samples, and 0/5 (0%) control samples. Median pericontusional Bcl-2 expression was significantly higher in craniotomy samples than in post-mortem samples (2 (range 0–2) versus 0 (range 0–1), *p* = 0.027). Bcl-2 expression in autopsy patients did not differ between pericontusional and distal zones. TUNEL-positive cells were seen in all samples of TBI patients who underwent surgical evacuation, 4 of 7 (57.1%) of post-mortem TBI patients, and none of the controls (*p* = 0.026) [15].

The largest histological study of Bcl-2 expression in TBI to date was published by Nathoo et al., in 2004 [16]. Tissue samples from the “peri-ischemic zone” of the white matter were obtained from 29 TBI patients (age 28.3 ± 15.3 years, 93.1% male) with supratentorial contusions. Patients had both moderate and severe injury (mean admission GCS 11.2 (range: 5–14)). Average time from injury to biopsy was 74.9 h (range: 7.8–289 h). TBI patients were compared to three controls who had undergone surgery for temporal lobe epilepsy. The authors assessed Bcl-2 expression by IHC and quantified expression by median maximum pixel density. Immunostaining detected Bcl-2 in 14 of 29 (48.3%) TBI patients with 2.9-fold increase when compared to epilepsy controls, but this was not statistically significant. There were increases of Bax (18-fold, *p* < 0.005) and caspase-3 (20-fold, *p* < 0.005) relative to controls. The mean GOS at 18 months was significantly higher in patients who were Bcl-2-positive than in patients who were Bcl-2-negative (*p* = 0.04). In a logistic regression model controlling for age, admission GCS, time to surgery, and contusion size, Bcl-2 negativity (odds ratio (OR) 5.5, 95% confidence interval (CI) 1.1–24.8, *p* < 0.04) and caspase-3 positivity (OR 1.4, 95% CI 1.1–1.8, *p* < 0.01) was an independent predictor of mortality at 18 months [16].

The most recent study examining histological expression of Bcl-2 after TBI was performed by Dai and colleagues [17]. They compared cortical tissue from 25 TBI patients to tissue from 30 patients undergoing surgery for glioma resection, although no other clinical or demographic information was detailed. The authors performed automated quantitation of immunohistochemistry to evaluate expression of Bcl-2 and Nur-77. Nur-77 is a transcription factor of the nuclear receptor family and is a putatitve regulator of Bcl-2 [18]. They found that expression levels of Bcl-2 and Nur-77 in TBI patients were both significantly upregulated (*p* < 0.001) and positively correlated (r = 1.051, *p* < 0.001) when compared to glioma patients, though these levels were non-zero in glioma. TUNEL assay also showed increased apoptosis in TBI patients relative to glioma patients (*p* < 0.001) [17].

Taken together, the above studies suggest that Bcl-2 is upregulated following contusional TBI in both adults and children at variable rates of expression. Bcl-2 is typically not expressed in the cortex of non-TBI patients post-mortem [12,15], after epilepsy resections [16], and near glioma margins [17]. Histological Bcl-2 positivity may be associated with lower mortality, although findings are limited by small sample sizes [14,16]. The presence of a dose-response relationship, and whether Bcl-2 positivity predicts functional outcome beyond mortality, remain uncertain.

### 3.2. Bcl-2 as a Biomarker in CSF and Serum After TBI

Clark and colleagues also performed the first study in TBI assessing CSF Bcl-2 protein expression [13]. The study included 23 pediatric severe TBI patients (60.9% male) and 19 pediatric controls (57.9% male) who had undergone lumbar puncture to rule out infection. CSF was collected over the first three days following injury from a continuously draining external ventricular drain (EVD), and Bcl-2 was assessed by Enzyme-Linked Immunosorbent Assay (ELISA). Bcl-2 was detectable in 21 of 23 (91.3%) TBI patients, and the average Bcl-2 was over three times higher in TBI patients than in controls (9.70 ± 1.43 versus 2.68 ± 0.85 U/mL, *p* = 0.01). In contrast to histological analysis, more controls had detectable levels of Bcl-2 in CSF. Bcl-2 levels did not correlate with CSF total protein (r = 0.025, *p* = 0.921), suggesting that CSF Bcl-2 expression is not an epiphenomenon of blood–brain barrier permeability. The Bcl-2 levels in the first three days after injury were 7.09 ± 1.84 U/mL on day 1, 10.58 ± 2.54 on day 2, and 11.77 ± 3.06 on day 3. Increased CSF Bcl-2 was associated with survival in univariable (*p* = 0.002) and multivariable (*p* = 0.018) models. As with histology, CSF Bcl-2 was not associated with patient age or initial GCS [13].

Two other studies assayed CSF for Bcl-2 protein expression without concomitant investigation of brain tissue. Uzan and colleagues longitudinally assessed CSF Bcl-2 levels on post-injury days 1–10 in a mixed cohort of 14 pediatric and adult severe TBI patients (78.6% male) [19]. CSF was collected from an EVD when ICP exceeded 15 mmHg, rather than continuously. They compared Bcl-2 levels in the CSF to those in control CSF taken from 14 adult and pediatric patients undergoing spinal anesthesia for non-spinal and non-cranial procedures (71.4% male). Unlike Clark et al. [13], Uzan and colleagues found that Bcl-2 was detectable at all time points in all TBI patients, but not in any of the control patients (*p* < 0.01) [19]. CSF Bcl-2 tended to increase over the first 5 days following injury, with a significant difference between days 1 and 2 (*p* < 0.05), and most frequently peaked on day 3 (119 ± 28.5 ng/mL), before declining after day 5 and returning by day 7 to levels comparable to day 1. CSF Bcl-2 was not associated with mean CPP or ICP (measured during the 8 h before CSF collection), or with severity of intracranial injury by Marshall classification [20]. Caspase-3, an executioner caspase in canonical apoptotic pathways [21], was detected in all TBI patients but in none of the controls (*p* < 0.01), and its level was highest on day 5 (3.8 ± 1.3 uM/min). Caspase-3 level was associated with ICP (*p* = 0.01) and CPP (*p* = 0.04) [19]. The association between biomarkers and GOS score was not conducted in the study.

In the largest study of CSF Bcl-2 expression following TBI, Wagner et al., trended Bcl-2 levels in 76 adult severe TBI patients (80% male) over the course of five days after injury by ELISA [11]. Levels of Bcl-2 in CSF, taken from a continuously draining EVD, were compared to levels in healthy controls. Bcl-2 levels were detectable in controls at baseline (5.82 ± 0.34 U/mL) but were significantly increased in TBI patients on days 1, 2, and 4 (*p* < 0.05). The investigators reported a nearly 1.5-fold difference in Bcl-2 levels between patients and controls. Trajectory analysis of CSF Bcl-2 showed three distinct groups: (1) Bcl-2 gradually rose until exceeding control levels by day 4 (riser group, 11%), (2) levels consistently remained near those of controls (low group, 31%), and (3) consistently higher than controls (high group, 58%). Patients in the “riser” group had the best outcomes at 6-month GOS (GOS 1: 0% riser, 20% low, 37% high; GOS 2/3: 17% riser, 30% low, 46% high; GOS 4/5: 83% riser, 50% low, 17% high; *p* = 0.009), 12-month GOS (GOS 1: 0% riser, 19% low, 47% high; GOS 2/3: 0% riser, 14% low, 27% high; GOS 4/5: 100% riser, 67% low, 27% high; *p* = 0.012), as well as 6-month DRS (*p* = 0.009) and 12-month DRS (*p* = 0.003). Multivariable regression showed that patients in the low and riser groups had better GOS (OR 8.9, 95% CI 2.3–34.4, *p* = 0.002) and DRS (OR 8.7, 95% CI 2.5–30.9, *p* = 0.001) than the high group at 12 months. These results were adjusted for age, GCS, hospital length of stay, and trajectory of cytochrome C [11].

One study to date has assessed Bcl-2 levels in the serum of TBI patients [22]. The authors used Bcl-2 as a surrogate outcome in a small trial where 40 patients with moderate (GCS 9-12) TBI were randomized to either standard care or adrenocorticotropic hormone (ACTH_4-10_Pro8-Gly9-Pro10), a synthetic ACTH analog. A 5-day treatment course of intranasal ACTH analog was administered in the treatment arm. Serum levels of Bcl-2 on days 1 and 5 by ELISA were measured, and outcomes on the Mini Mental State Examination (MMSE) and Barthel Index at discharge were analyzed. Details of the methodology on patient screening, randomization/blinding, the reference level utilized in the assays, or any adverse events were not provided. There was no change in mean serum Bcl-2 between days 1 and 5 in the standard care arm (1.68 ± 1.34 ng/mL versus 1.66 ± 1.06 ng/mL), although there was notable inter-individual variability. In the treatment arm, serum Bcl-2 increased in 17 of 19 (89.5%) patients, from 1.93 ± 1.35 ng/mL on day 1 to 3.81 ± 1.00 ng/mL on day 5 (*p* < 0.05). The study ultimately found no association between Bcl-2 level in serum and MMSE or Barthel Index score [22]. The statistical validity to the findings was not clear given the limitations stated above, and validation of serum Bcl-2 as an intermediate outcome is lacking in the literature.

In agreement with key findings on Bcl-2 expression in the cortical tissue, biomarker analyses of CSF Bcl-2 show upregulation in TBI patients when compared to controls [11,13,19]. Inter-study differences in CSF Bcl-2 levels of controls may be related to differences in assay or study design. Bcl-2 in CSF in general increases over the first 5 days following injury, before returning baseline after head injury [11,19]. While expression of Bcl-2 in histological sections was almost uniformly associated with improved survival, temporal profiles of Bcl-2 in CSF are more complex and are associated with variable functional outcomes.

### 3.3. Genetic Factors Associated with Human BCL2

**Figure 2:**
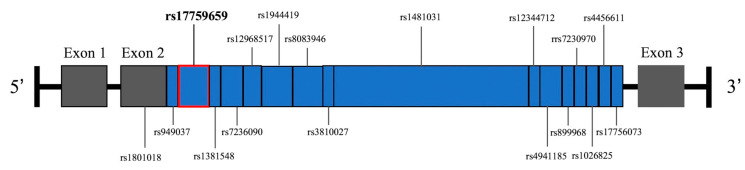
Map of the *Bcl-2* gene on chromosome 18q21.3.

To date, only one study has assessed the impact of genetic variability within the BCL2 gene on outcomes following TBI [23]. Using a tag single nucleotide polymorphism (SNP) approach, Hoh et al., studied a cohort of 205 adult severe TBI patients (80% male). Although the initial study enrolled non-Caucasian patients, they excluded all non-Caucasian patients from the analysis given low racial diversity. They studied 17 tag SNPs with minor allele frequency ≥ 30% and r^2^ ≥ 0.80 located throughout the BCL2 gene. The outcomes of interest were 3-month mortality, and longitudinal functional outcome (GOS and DRS) and neurobehavioral outcomes (neurobehavioral rating scale-revised (NRS-R)) over 3–24 months. No SNPs were significantly associated with mortality, DRS, or NRS-R. The variant allele of *rs17759659* is found in intron 2 of the BCL2 gene with wildtype genotype AA and variant genotypes AG and GG (Figure 2). Compared to wildtype allele A, the presence of the variant allele G was associated with greater mortality (OR 4.23, 95% CI 1.31–13.61, *p* = 0.02). The variant allele was also associated with poorer outcomes on the GOS (*p* = 0.001) and DRS (*p* = 0.002), with GOS remaining significant after Bonferroni correction (*p* ≤ 0.001). Molecular and physiological correlation were not investigated in this analysis to better elucidate mediators of observed genotype–phenotype association. Limitations included low female enrollment and the lack of non-Caucasian participants.

### 3.4. Studies of Bcl-2 in In-Vitro TBI Models Using Human Cell Lines

The studies by Miñambres et al., and Dai et al., included further in-vitro work [15,17]. As part of their larger study of severe TBI patients, Miñambres et al., collected serum from their patients at 48 h following intensive care unit (ICU) admission [15]. Pheochromocytoma (PC12) cells were exposed to heat-inactivated serum of 5 TBI patients who also gave tissue. They observed that the proportion of apoptotic cells following exposure to the serum of 2 Bcl-2-positive patients was uniformly lower than the proportion of apoptotic cells following exposure to the serum of 3 Bcl-2-negative patients (median 64.4%, range 62.8–66% versus median 73.8%, range 73.5–74.9%). Increased induction of apoptosis from TBI serum exposure was also associated with increased 6-month mortality for the patients (OR 1.95, 95% CI 1.14–3.32, *p* = 0.014) [15].

Dai and colleagues also performed in-vitro work with PC12 cells, though they did not first induce differentiation [17]. They describe dividing cells into four experimental groups: negative control, TBI model, TBI + cyclosporine A (Nur77 inhibitor), and TBI + APG-1252 (Bcl-2 inhibitor). Their TBI model consisted of using a micropipetter with a 200 µL tip to detach the cells. Levels of expression of Nur-77, Bcl-2, cytochrome C, and caspase-3 were measured in each group. Compared to the negative control, the TBI model had increased expression of all four proteins (*p* < 0.001). While all of the protein levels in the TBI + cyclosporine A group returned to control levels, in the TBI + APG-1252 group, a persistent Nur-77 upregulation was observed (*p* < 0.01), which was suggestive of apoptotic regulation by Nur-77 upstream of Bcl-2 after TBI [17]. These studies suggest that Bcl-2 is part of a larger apoptosis-regulating network, and the regulatory role of Bcl-2 likely depends on other factors in the posttraumatic milieu.

## 4. Discussion

Head trauma activates the apoptotic cascade in neurons and glia as part of secondary cellular injury. Improved knowledge on this topic has obvious clinical implications for monitoring, treatment, and prognostication [24,25]. Apoptosis, also known as programmed cell death (PCD), is distinct from necrosis—the consequence of mechanical injury—as an active and genetically driven process that in general involves the caspase family proteases [26,27]. Caspase-dependent PCD occurs via either intrinsic (induced by stress on cellular organelles) or extrinsic (cell surface coupling to form death-inducing signal complexes) pathways [28,29]. The phenotypic features of apoptotic neuronal loss are cellular shrinkage, DNA fragmentation, and formation of apoptotic bodies [30,31]. Although it was first described in the 1940s [32], only in the past two decades has there been human research on apoptosis and its various key regulators after TBI.

### 4.1. Bcl-2 Expression and Association with Neurological Outcomes

B-cell lymphoma 2 (Bcl-2) is a proto-oncogene that was first identified in follicular lymphoma [33]. The anti-apoptotic BCL2 gene and its 26 kDa protein protect cells from traumatic and ischemic stimuli that induce apoptotic cell death. It is found intracellularly near sites of free radical generation, including the mitochondria, endoplasmic reticula, and nuclear membranes, to prevent peroxidation injury [34,35,36]. Other mechanisms include the maintenance of calcium homeostasis [37,38], heterodimerization with Bax [39,40], and inhibiting mitochondrial release of pro-apoptotic proteins, including cytochrome *c* and endonuclease G [41,42,43,44]. The Bcl-2 family proteins regulate membrane permeability through highly conserved homology domains that are important for homo- and hetero-complex formation and pore formation [7,45]. The formation of these complexes can result in the release of apoptotic proteins from the mitochondria after TBI in rats [10]. The anti-apoptotic Bcl-2 protein can inhibit pore formation and mitochondrial release; thereby, in human subjects, BCL-2 and its gene products are found to be upregulated in injured cortex and associated with cells that were able to survive, as well as being associated with more favorable outcomes after TBI [13,14]. In experimental models of the nervous system, *Bcl-2* mRNA expression and Bcl-2 protein are found to be increased in surviving neurons [10,45,46,47,48].

The current evidence on Bcl-2 in TBI patients mainly arises from pericontusional immunohistochemistry and analysis of CSF biomarkers. There is upregulation of Bcl-2 at translational levels in the peritraumatic cortex of TBI patients obtained from decompressive craniectomies. In cohorts of primarily severe TBI patients, Bcl-2 is detected in 36% to 75% of patients, while it is not present in most of the non-TBI controls [12,16]. In comparison with non-TBI neurosurgical controls, i.e., patients undergoing epilepsy and glioma resections, Bcl-2 expression is greater after head injury, ranging from 2.9-fold to 17-fold [15,16,17]. Notably, two studies, Ng et al., and Nathoo et al., demonstrated that mortality was lower for Bcl-2-positive TBI patients relative to Bcl-2-negative TBI patients at 6- and 18-month follow-up, respectively [14,16]. Evidence so far is in agreement with the anti-apoptotic role of Bcl-2 in the peritraumatic cortex, however validation from larger sample sizes is necessary and underway. Increased understanding of the biomolecular role of Bcl-2 has allowed for investigations into novel therapeutic opportunities, particularly in the treatment of cancer [49,50]. Bcl-2 studies in TBI patients are currently in the early phase, and challenges of targeted modulation include safety and efficacy of Bcl-2 mimetic protein delivery in human subjects in comparison to mice models [51]. There is potential to not only improve injury stratification, but also to reduce secondary injury burden of patients after presenting with brain injury.

In rodent models, Clark et al., found that *Bcl-2* mRNA is induced 6 h after initial injury in the ipsilateral pericontusional cortex, followed by a rise in Bcl-2 protein at 8 h that is maintained up to 7 days [10]. Investigations utilizing CSF biomarkers have been able to provide similar temporal profiles in humans, where Bcl-2 is typically a membrane-bound protein that is minimally detectable in the postnatal brain tissue and CSF. After sustaining a head injury, however, it is induced in both pediatric and adult patients [13]. Bcl-2 levels in the CSF begin to increase on post-injury day 1, peak on days 3 and 4, then gradually decline after day 5 [19]. Changes in the CSF milieu can closely reflect the intracranial response to injury, correlating with histological studies that use injured cortical tissue, but with the additional benefits of accessibility and the therapeutic potential of intracranial monitoring. Clark et al., reported greater likelihood of survival with increased CSF Bcl-2 after TBI [13]. More specifically, Wagner et al., identified better GOS outcomes with the classic rise in Bcl-2 profile as compared to worse outcomes with persistent elevations in Bcl-2 [11]. Reasons for this discrepancy are difficult to discern without a better understanding of which cell types are responsible for expressing the Bcl-2 being measured in CSF, and how much Bcl-2 is being released by the nervous system versus infiltrating inflammatory cells. One explanation is that upregulation of Bcl-2 occurs in the days following injury, and is associated with neuroprotection and improved outcomes, whereas early and persistent Bcl-2 expression is more indicative of inflammatory burden and injury severity.

### 4.2. Apoptotic Cell Death in the Peritraumatic Cortex

Apoptosis is characterized by morphological and biochemical features, including the presence of apoptotic bodies, internucleosomal fragmentation, and apoptotic gene products. It was only recently that we began to confirm apoptosis in the contusional tissue of patients after trauma. TUNEL assays for DNA fragmentation and damage are suggestive of apoptotic cell death [10,49]. Several studies have utilized TUNEL labeling to identify late stages of cellular apoptotic activity present near margins of traumatic contusions, in comparison to specimens from epilepsy lobectomies [16], glioma resections [17], and non-TBI post-mortem autopsies [12,15]. It is logical to demonstrate that pericontusional regions vulnerable to post-traumatic insults are frequently TUNEL-positive and demonstrate morphological features of apoptotic cell death, in contrast to most non-TBI controls that are TUNEL-negative. A limitation of TUNEL analysis is specificity to apoptosis versus necrosis from mechanical injury. Findings from rodent models have shown that Bcl-2 protein is mostly expressed in surviving neurons that may be vulnerable to injury, and importantly, these are TUNEL-negative neurons without morphological or biochemical evidence of apoptosis [10]. In these experimental models, the timeframe of TUNEL-positive cells also coincides with increases in Bcl-2 protein levels, suggesting that neurons at risk for apoptosis but expressing Bcl-2 can be protected from endonucleases and nuclear damage.

### 4.3. Other Regulatory Proteins of Apoptosis

A number of pro-death proteins have also been investigated in parallel with Bcl-2 using injured brain tissue samples. Bax is a 21 kDa protein and an apoptotic promoter by increasing cellular sensitivity to apoptotic stimuli and accelerating cell death [39]. Bax expression in human brain tissue is variable, with several studies reporting no significant difference between TBI patients and non-TBI controls in regards to the expression of Bax [12,14,15]. Nathoo et al., however, did find that Bax expression was upregulated 18-fold in pericontusional tissue when compared to epilepsy controls [16]. Bax is an early responder to trauma and its expression is easily detectable with a half-life of 4–24 h [50]. The tumor suppressor p53, with a short half-life of 15 min, induces Bax and suppresses Bcl-2 expression in rats [51,52]. For these reasons, Bax and p53 may be part of a conserved mechanism in response to injury, therefore the presence of Bax or absence of detectable levels of p53 are not associated with clinical outcomes. Rather, Bcl-2 expression and the interaction between Bcl-2 and apoptotic promoters are potentially more important to risk-stratification and prognostication during the clinical assessment of TBI patients.

Bcl-x_L_ is another anti-apoptotic protein that in experimental models is shown to inhibit the release of cytochrome *c*, endonuclease G, and Apoptosis Inducing Factor (AIF) from the mitochondria [43,53,54]. Current findings on increased Bcl-x_L_ expression in the pericontusional cortex of injured patients are equivocal. Clark et al., did not measure differences in relative levels of Bcl-x_L_ between TBI and control patients [12], but Miñambres and colleagues showed increased Bcl-x_L_ expression in TBI patients, as well as greater rate of apoptosis in cells that are cultured with low levels of Bcl-x_L_ [15]. Cross-talk occurs between the agonists and antagonists of PCD [55], therefore it is important to recognize that in addition to altered expression levels, interactions that result in protein translocation and activation also contribute to apoptosis.

### 4.4. Genetic Variability of the Bcl-2 Gene

There is a growing body of literature investigating genetic factors associated with variability in secondary neurochemical and pathophysiological changes after TBI [55,56,57,58,59,60]. However, studies on the genetic variability of pro-survival Bcl-2 expression remain unclear. The BCL2 gene is located on chromosome 18q21.3, and it includes three exons (Figure 2). In a candidate gene study of 17 BCL-2 SNPs, we have identified the SNP *rs17759659*, an intronic SNP near the 5′ end of intron II, as a predictor of 3-month functional outcomes [23]. Blood samples were collected via peripheral venipuncture or existing arterial catheters within 24 h of presentation of patients with severe TBI at our institution. Recently, genotyping from these biospecimens showed that the presence of both *rs17759659* variant alleles carry greater risks of intracranial hypertension during the 5 days post trauma, edema, and the need for surgical intervention [61]. Possible mechanisms may be increased risk of intracranial hypertension and development of cerebral edema as a result of apoptosis activation due to variable levels of Bcl-2 protein expression. While the study was the first to identify the clinical relevance of rs17759659, it has since been associated with lymphocyte apoptosis in autoimmune thyroiditis in an eastern European population [62], and with endometrial cancer risk in a Chinese population [63]. Research on this topic in the TBI population is ongoing and can provide further supportive evidence.

## 5. Limitations

Research conducted to date on Bcl-2 expression and apoptosis in patients experiencing TBI remains low in number and sample size for various reasons, which have been mentioned previously. The deficiency of large controlled cohort studies in the literature is a significant limitation to the breadth of our analysis. As such, this reflects the critical need to design additional prospective controlled studies to generate Class II evidence on this topic. The frequent obstacles in TBI research are the heterogeneity of injuries and institutional variability in terms of clinical practices. Studies thus far consist mainly of immunohistological analysis that confirmed Bcl-2 upregulation in pericontusional regions of the brain post trauma. An imminent future direction is defining the relationship between Bcl-2 upregulation and secondary injury burden post trauma, which include clinical and radiographic data that are obtained during the acute management of TBI. Measurement of Bcl-2 expression in patients has the methodological challenge of obtaining tissue samples near injured cortical regions, which is not the case in experimental animal models. With the exception of CSF and serum studies, most specimens are collected at various post-injury times when surgical evacuation is needed. In spite of this, there is emerging evidence on the role of biomarkers in order to individualize TBI management and prognostication. Research on Bcl-2 and apoptosis can further benefit from improved understanding of the impact of genotype variability on clinical outcomes, which will constitute another imminent future direction to identify targeted genotype-based therapies for subpopulations of TBI patients.

## 6. Conclusions

Outcomes after TBI depend on the extent of the primary traumatic insult and dynamic changes in the secondary pathophysiology. Improved knowledge of Bcl-2 and its regulatory role on apoptosis can aid clinical assessment and the development of targeted therapies to suppress secondary neuronal loss. In neurons vulnerable to programmed cell death, increased levels of Bcl-2 exert a neuroprotective role and show promise as a biomarker with diagnostic capability. Bcl-2 is upregulated in the peritraumatic cortex of adults and pediatric patients, particularly after sustaining a survivable head trauma and with increased circulating Bcl-2 in the CSF in the acute post-injury period. Evaluation of variability in the BCL2 gene, Bcl-2 protein level, and the development of secondary pathophysiology can enhance the current management of patients who sustained TBI.

## Figures and Tables

**Table 1:** Summary of investigations on Bcl-2 and apoptosis after TBI in human subjects.

Author and Year	Study Type	N	Sample Characteristics	Description	Methodology	Results
**Pericontusional Tissue Analysis**						
Clark et al., 1999 [12]	Prospective cohort	8 TBI adults (6 male, 2 female), 6 non-TBI controls (2 male, 4 female)	Pericontusional tissue of adults who underwent decompressive craniectomy and surgical resection	PCD cascade activation and neuronal expression of Bcl-2	Immunohistochemistry and expression of Bcl-2, Bcl-x_L_ and Bax, and cleavage of caspase-1 and caspase-3. Detection of TUNEL-positive apoptotic cells.	Compared to non-TBI controls, TBI patients had increased Bcl-2 expression (17-fold, *p* = 0.020). Pro-caspase-1 showed 2-fold reduction, while the p10 fragment of caspase-1 was increased (78-fold increase, *p* < 0.001). Caspase-3 is upregulated 14-fold (*p* = 0.020) to permit formation of active enzyme complexes. TUNEL-positive cells were detected in most TBI samples and in 2/6 non-TBI controls.
Ng et al., 2000 [14]	Prospective cohort	11 severe TBI adults (8 male, 3 female)	Pericontusional tissue (7 frontal, 4 temporal, 4 parietal) of adults who underwent craniotomy and surgical resection for mass effect	PCD cascade activation and Bcl-2 expression after severe TBI	Immunohistochemistry and expression of Bcl-2, Bax, and p53. Detection of TUNEL-positive apoptotic cells.	Bcl-2 was detected in 6/11 (55%) samples. Notably, 4/5 (80%) of patients without Bcl-2 expression had time of trauma to surgery ≤8 h, versus 2/6 (33%) of patients positive for Bcl-2 expression. ICPs were 13.5 ±3.72 mm Hg in Bcl-2-positive patients versus 40.8 ± 30.28 mm Hg in *Bcl-2*-negative patients (*p* = 0.057). 6/6 Bcl-2-positive patients were alive at 6 months compared to 1/5 Bcl-2-negative patients (*p* = 0.01). TUNEL-positive cells were detected in 8 (73%) of the 11 patients.
Nathoo et al., 2004 [16]	Prospective cohort	29 moderate and severe TBI (27 male, 2 female), 3 epilepsy-surgery controls	Pericontusional tissue of adults who required emergency craniotomy for supratentorial pathology	Identify evidence of apoptosis associated with traumatic cerebral contusions and correlation with clinical outcomes	Immunohistochemistry of apoptosis-related cell proteins Bcl-2, p53, Bax, and caspase-3	There were increases of Bax (18-fold; *p* < 0.005) and caspase-3 (20-fold; *p* < 0.005), whereas Bcl-2 was upregulated in only 14 patients (48.3%; 2.9-fold increase) compared with control tissue. Bcl-2-positive patients experienced improved outcome on GOS when compared with the Bcl-2-negative patients at 18 months of follow up (*p* = 0.03), despite having a higher mean age and lower admission GCS scores. Regression analysis found Bcl-2-negative status (*p* < 0.04, OR 5.5; 95% CI 1.1–28.4) and caspase-3-positive status (*p* < 0.01, OR 1.4, 95% CI 1.1–1.8) as independent predictors of poor outcome.
Miñambres et al., 2008 [15]	Prospective cohort and in vitro	11 severe TBI adults (8 male, 3 female), 5 non-TBI controls (4 male, 1 female)	Pericontusional tissue obtained from surgical resection (4) or post-mortem via autopsy (4)	PCD cascade activation, Bcl-2 expression, and in vitro neuronal apoptosis (PC12 cells)	Immunohistochemistry and expression of Bcl-2, Bcl-x_L,_ Bim, Bax, and Fas. Detection of TUNEL-positive apoptotic cells. In vitro apoptosis induced by TBI patients’ serum	Bcl-2 (4/11 versus 0/5) and Fas (6/11 versus 0/5) were only immunoreactive in TBI patients. Compared to controls, Bcl-2 expression was higher in craniotomy group (*p* = 0.027), Fas was higher in both craniotomy (*p* = 0.09) and post-mortem (*p* = 0.007) groups, and Bcl-x_L_ was lower in post-mortem group (*p* = 0.014). Anti-apoptotic Bcl-2 (*p* = 0.027) and Bcl-x_L_ (*p* = 0.014) were higher in the emergency craniotomy cohort relative to post-mortem TBI patients. TUNEL-positive cells were detected in 4/4 samples of craniotomy cohort, 4/7 (57%) of post-mortem cohort, and 0/5 of controls (*p* = 0.026). There was greater early apoptosis in the cultures of PC12 induced by the serum of patients with low Bcl-2 and Bcl-x_L_ levels (median 64.4% versus 73.8%), and with non-survivors.
Dai et al., 2018 [17]	Prospective and in vitro	30 patients with glioma and 25 TBI patients of unknown severity	Glioma tissues from biopsy/resection and cerebral tissues from TBI patients were collected	Determine the mechanism by which Nur77 and Bcl-2 protein expression influence apoptosis after TBI	Nur77 and Bcl-2 expression by IHC assay and immunofluorescence. Detection of TUNEL-positive apoptotic cells. Nur77 inhibitor via injection with 1 mL/kg CsA, and Bcl-2 inhibitor using 1 mL/kg APG- 1252	Apoptotic cells are increased in TBI cohort compared to glioma group (*p* < 0.001). Nur77 and Bcl-2 expression is upregulated after TBI (*p* < 0.001), and there was a positive correlation between Nur77 and Bcl-2 in TBI tissues (r = 1.051, *p* < 0.001). Nur77 play a promoting factor in nerve cell apoptosis-induced TBI via Bcl-2/Cyto C/Caspase 3 in vitro and vivo. Bcl-2 may promote apoptosis in some cases, acting as a pro-apoptotic protein.
**CSF Biomarker Analysis**						
Clark et al., 2000 [13]	Prospective cohort	23 severe TBI pediatric patients (14 male, 9 female), 19 non-TBI controls (11 male, 8 female)	CSF samples collected on days 1, 2, and 3 after TBI, and brain tissue of 2 patients who needed decompressive craniectomy and surgical resection	PCD cascade activation, Bcl-2 expression, and DNA degradation in infants and children	Levels of Bcl-2 and oligonucleosomes in CSF. Detection of TUNEL-positive apoptotic cells.	Mean CSF Bcl-2 concentrations were increased in patients after TBI compared with control (9.70 ± 1.43 versus 2.68 ± 0.85 U/mL, *p* = 0.01). Increased CSF Bcl-2 was independently associated with patient survival on multivariate analysis (*p* = 0.018). CSF oligonucleosome concentration increased after TBI compared with control (428 ± 77 versus 168 ± 52 mU/mL, *p* = 0.08) and did not correlate with CSF Bcl-2 (r = –0.015, *p* = 0.905).
Uzan et al., 2006 [19]	Prospective cohort	14 patients with severe TBI (11 male, 3 female), 14 controls without TBI or spinal pathology	CSF samples drained on days 1, 2, 3, 5, 7, and 10 from pediatric (5) and adult (9) patients	Determine if soluble Bcl-2, Fas and caspase-3 would be increased in CSF after severe head injury	Bcl-2, sFas, and caspase-3 were measured in drained CSF samples after severe TBI. The concentrations of Bcl-2 were analyzed via ELISA	No Bcl-2, Fas, or caspase-3 were detected in CSFof controls, while levels were higher in CSF of patients at all time points post-trauma (*p* < 0.01). Peak Bcl-2 levels varied by individual, but frequently on days 3 and 4 (7 patients). Mean peak bcl-2 concentration was noted on day 3 (119 ±28.5 ng/mL) and declined after day 5. Bcl-2 levels in CSF did not correlateto ICP (*p* = 0.9), CPP (*p* = 0.7) and initial CTfindings (*p* = 0.4).
Hoh et al., 2010 [23]	Prospective cohort	205 subjects (163 male, 42 female) with severe TBI aged 16–75 years old	DNA was extracted from CSF or blood specimens for genotyping of regions within and around the *Bcl-2* gene.	Investigate if variation in the *Bcl-2* gene contributes to variability in the outcomes attained after severe TBI	All of the genetic variability associated with the *Bcl-2* gene were characterized utilizing 17 tSNPs. The GOS, DRS, and NRS-R scores were conducted at 3, 6, 12, and 24 months post-TBI.	The variant allele of *rs17759659* was associated with poorer outcomes (GOS, *p* = 0.001; DRS, *p* = 0.002), higher mortality (*p* = 0.02; OR = 4.23; CI 1.31–13.61), and worse NRS-R scores (*p* = 0.05). The variant allele for rs1801018 was associated with poorer outcomes (GOS, *p* = 0.02; DRS, *p* = 0.009), and mortality (*p* = 0.03; OR = 3.86; CI 1.18–12.59). Other polymorphisms including *rs7236090* and *rs949037* were associated with variable outcomes on the NRS-R, and DRS, although the only finding that stood up to Bonferroni correction was *rs17759659* for GOS.
Wagner et al., 2011 [11]	Prospective cohort	76 severe TBI patients (61 male, 15 female) aged 16–65 years old, 10 healthy adult control subjects	CSF samples for biomarker analysis from EVDs were collected for up to 6 days after initial trauma. Control subjects’ CSF was obtained via lumbar puncture	Bcl-2 and cytochrome C levels over time may reflect cellular injury response and predict long-term outcomes after TBI	CSF Bcl-2 and CytoC concentrations were measured daily from day 1 to 6 after injury via ELISA. GOS score and Disability Rating Scale (DRS) at 6- and 12-month follow-up were the primary outcome measures	Bcl-2 levels are significantly higher than controls on days 1, 2, and 4 (*p* < 0.05 all comparisons) and trended toward significance on day 3 (*p* = 0.051). CytoC levels peaked 24 h after injury and were higher than controls for days 1 to 4 (*p* < 0.043 all comparisons) and trended toward significance on day 0 (*p* = 0.074). Bcl-2 temporal profiles were categorized as riser (11%), low (31%), and high (58%). The combined low and riser Bcl-2 groups were significantly associated with better GOS-6 and GOS-12 scores *(p* = 0.009 and *p* = 0.002, respectively) when compared with the high Bcl-2 group. Patients in the low and riser Bcl-2 groups were 9 times more likely to have better outcome 12 months after injury.
**Other**						
Indharty et al., 2013 [22]	Prospective randomized trial	40 moderate TBI adults aged 18–29 years old	Standard therapy versus standard therapy plus intranasal ACTH in patients with contusion on CT scan without the indication for surgery	ACTH_4-10_Pro^8^-Gly^9^-Pro^10^ as a synthetic peptide constituting a short fragment of ACTH to potentially inhibit apoptosis by increasing Bcl-2 while minimizing hormonal side effects	5-day course of intranasal ACTH_4-10_Pro^8^-Gly^9^-Pro^10^ (Semax^®^): 9 mg/day 1, 6 mg/day 2, and 3 mg daily for the remaining 3 days. Blood draws on day 1 and day 5 to quantify Bcl-2 via ELISA	In the control group, mean Bcl-2 on day 1 was 1.68 ± 1.34 ng/mL and at day 5 was 1.66 ± 1.06 ng/mL. In the intervention arm, serum Bcl-2 level increased from day 1 1.93 ± 1.35 ng/mL to 3.81 ± 1.00 ng/mL on day 5 (*p* < 0.05), which was also significant on intergroup comparison (*p* < 0.05). There was no difference in clinical outcomes by the Barthel Index or MMSE, but there was a trend toward shorter hospital stay within the ACTH_4-10_Pro^8^-Gly^9^-Pro^10^ intervention cohort.

TBI = traumatic brain injury; PCD = programmed cell death; Bcl-2 = b-cell lymphoma 2; TUNEL = terminal deoxynucleotidyl transferase-mediated biotinylated dUTP nick-end labeling; GOS = Glasgow Outcome Score; GCS = Glasgow Coma Scale; CI = confidence interval; CSF = cerebrospinal fluid; DNA = deoxyribonucleic acid; tSNP = tagging single nucleotide polymorphism; DRS = Disability Rating Scale; Neurobehavioral Rating Scale-Revised; ACTH = adrenocorticotropic hormone; MMSA = Mini-Mental State Exam; ELISA = enzyme-linked immunosorbent assay.
